# Structure to function analysis with antigenic characterization of a hypothetical protein,HPAG1_0576 from Helicobacter pylori HPAG1

**DOI:** 10.6026/97320630015456

**Published:** 2019-07-31

**Authors:** Hanan Ashrafi, Muntequa Ishtiaq Siraji, Nazmir Nur Showva, Md. Mozamme Hossain, Tareq Hossan, Md. Ashraful Hasan, Abdullah Mohammad Shohael, Mohammad Mahfuz Ali Khan Shawan

**Affiliations:** 1 Department of Biotechnology and Genetic Engineering, Jahangirnagar University, Savar, Dhaka-1342, Bangladesh; 2 Department of Biochemistry and Molecular Biology, Jahangirnagar University, Savar, Dhaka-1342, Bangladesh; 3 Department of Biomedicine,University of Bergen, Bergen, Norway; 4 Department of Genetic Engineering and Biotechnology, University of Dhaka, Dhaka,Bangladesh

**Keywords:** H.pylori HPAG1, hypothetical protein, B and T cell epitopes, drug target, vaccine candidates

## Abstract

Helicobacter pylori, a unique gastric pathogen causing chronic inflammation in the gastric mucosa with a possibility to develop gastric cancer has one-third of its proteins still uncharacterized. In this study, a hypothetical protein (HP) namely HPAG1_0576 from H. pylori HPAG1 was chosen for detailed computational analysis of its structural, functional and epitopic properties. The primary, secondary and 3D structure/model of the selected HP was constructed. Then refinement and structure validation were done, which indicated a good quality of the newly constructed model. ProFunc and STRING suggested that HPAG1_0576 shares 98% identity with a carcinogenic factor, TNF-α inducing protein (Tip-α ) of H. pylori. IEDB immunoinformatics tool predicted VLMLQACTCPNTSQRNS from position 19-35 as most potential B-cell linear epitope and SFLKSKQL from position 5-12 as most potent conformational epitope. Alternatively, FALVRARGF and FLCGLGVLM were predicted as most immunogenic CD8+ and CD4+ T-cell epitopes respectively. At the same time findings of IFN epitope tool suggests that, HPAG1_0576 had a great potential to evoke interferon-gamma (IFN-γ) mediated immune response. However, this experiment is a primary approach for in silico vaccine designing from a HP, findings of this study will provide significant insights in further investigations and will assist in identifying new drug targets/vaccine candidates.

## Background

A newly sequenced bacterial genome usually consists of 30-40% genes that do not have known functions [1].A group of proteins is encoded by a substantial part of these genes whose translation has not been demonstrated and no experimental chemical evidence has been found. This group is defined as hypothetical proteins [2]. Despite of not being characterized; elucidation of their structural and functional secrets may reveal new domain and motifs, pathways and cascades, structural conformations and protein networks etc. [3]. Furthermore, novel HPs may also serve as pharmacological targets [4]. One of the most challenging problems in post genomic era is to determine protein functions due to the cost and time requirements for experimental approaches. Moreover, the high percentage of HPs in a genome makes their annotation even more difficult. This leaves bioinformatics with the opportunities to annotate protein functions by efficient, automated methods which are based on several algorithms and database of experimentally determined proteins [5,6].

Helicobacter pylori, a gram-negative bacterium has been classified as the definitive carcinogen of human gastric cancer and it is the fourth most prevailing cancer in the world. Infection with H. pylori induces chronic gastritis, peptic ulcer, mucosa-associated lymphoid tissue lymphoma and finally stomach cancer. Common virulence factors involved in these events are genes for cag Pathogenicity Island (cagA), vacuolating cytotoxin (vacA) and blood group antigen binding adhesions (babA and sabA). But the induction of proinflammatory cytokines such as IFN-γ, TNF-α, IL- 6 and IL-8 during H. pylori infection indicates the existence of unique virulence factors that play a vital role in the prognosis of inflammation to carcinogenesis [7]. Such a protein, TNF-α inducing protein (Tip-α) has been identified as a new carcinogenic factor of H. pylori. It is a 19 κDa protein and released as a homodimer from H. pylori and dimer formation is must for its cancerous activity [8]. This current study aimed to identify a novel virulent factor from the HPs of H. pylori HPAG1 and ultimately found a member of Tip-α family (HPAG1_0576). This strain of H. pylori was targeted because among the 1536 proteincoding genes, around 500 were found as hypothetical (till July, 2016) according to the information obtained from NCBI and KEGG database.

Tip-α is found only among H. pylori gene products with no obvious homolog in other species. To investigate the mechanism of a protein that is like Tip- α, it was necessary to establish the structure-function relationship [8]. In this study, the 3D structure of HPAG1_0576 was predicted by homology modeling and later was used for screening and designing new compound leading to the development of novel therapeutic strategy [9]. In addition, primary and secondary sequence/structure analyses, functional annotation, binding site prediction, PPI network generation were also performed. The study further attempted to combine best in silico approaches to identify potential epitopes that have high affinity for human MHC I and MHC II molecules, as well as to evaluate the IFN-γ inducing effect of HPAG1_0576; a critical step in the development of vaccines. The findings of this experiment will be very helpful for better understanding the disease mechanism and find novel drug targets with effective vaccine candidate to combat against H. pylori.

## Methodology

### Protein selection and sequence retrieval:

For the selection of protein, KEGG gene database (https://www.genome.jp/kegg/) was searched for HPs of H. pylori HPAG1 [10,11]. The search result identified 494 HPs along with their sequences and a combined protein blast (blastp) was performed with those sequences [12,13]. Based on the prediction outcomes the best scoring protein, HPAG1_0576 (NCBI protein id ABF84643) was selected for this study. The protein sequence of the selected HP was obtained from UniProtKB (https://www.uniprot.org/) in FASTA format (UniProtKB accession no Q1CTS9) [14-16].

### 

Primary and secondary structure prediction: The primary structure of the target protein was analyzed by ExPASy ProtParam tool (http://web.expasy.org/protparam/) which computes physicochemical properties such as, molecular weight, theoretical pI, instability index, aliphatic index, amino acid composition, extinction coefficient and grand average of hydropathicity (GRAVY) [17]. The secondary structure was scrutinized by self-optimized prediction method with alignment (SOPMA) (https://prabi.ibcp.fr/htm/site/web/) [18].

### Homology modeling:

An automatic modeling tool, Phyre2 (http://www.sbg.bio.ic.ac.uk/phyre2) was used to predict the 3D models of the target protein. It also predicts secondary structure, disorder and structural alignment for the submitted protein sequence [19]. Superimposition of the best protein model with its template was performed by RaptorX server (http://raptorx.uchicago.edu/) [20].

### Model validation:

The best model generated by Phyre2 was refined by ModRefiner (https://zhanglab.ccmb.med.umich.edu/ModRefiner/) which removes local distortion [21]. After that, accuracy and stereo chemical quality of the model were checked by PROCHECK (http://www.ebi.ac.uk/pdbsum) [12]. The overall quality of the refined structure was validated by Verify3D (http://services.mbi.ucla.edu/Verify_3D/) [22] and ERRAT analysis (http://services.mbi.ucla.edu/ERRAT/) [23]. QMEAN (https://swissmodel.expasy.org/qmean/cgi/index.cgi) [24] and ProsA (https://prosa.services.came.sbg.ac.at/prosa.php) [25] web servers were used to evaluate energy profile and verify structure in terms of Z score. To facilitate visualization, PyMOL was used to view both the energy minimized and superimposed structures [26].

### Function prediction from 3D structure:

An independent server, ProFunc (http://www.ebi.ac.uk/thornton-srv/databases/ProFunc/) was used to identify the probable functions of the target protein, which considered 3D structure as input and utilizes a combination of sequence and structure based approaches such as InterProScan, blast vs PDB, superfamily search, SSM fold match, 3D template search for enzyme, reverse templates and DNA/ligand binding sites etc. [27].

### Determination of Protein-Protein Interaction (PPI):

In this study, STRING 9.05 was used to search the interacting partners of the target protein. Predicted interactions were sorted by scores such as low confidence scores <0.4; medium, 0.4 to 0.7 and high >0.7 (http://string-db.org) [28].

### Prediction of binding sites and druggable pockets:

Shape and size parameters of protein pockets and cavities are important for active site analysis and structure-based ligand design. In this experiment, computed atlas of surface topography of proteins (CastP) (http://sts.bioe.uic.edu/castp) was used to identify probable binding sites, pockets and cavities from the 3D structure of the target protein [29].

### Determination of antigenicity and prediction of epitopes:

The amino acid sequence of the target protein was subjected to VaxiJen server (http://www.ddgpharmfac. net/vaxijen/VaxiJen/VaxiJen.html) [13], which determines its antigenic property at threshold 0.4. NetCTL1.2 server (http://www.cbs.dtu.dk/services/NetCTL/) was used to predict CD8+ T cell epitopes at a threshold of 0.75, which execute MHC class I binding prediction of epitopes to 12 MHC supertypes. The interacting alleles (MHC I-binder) with these epitopes were then identified by Immune Epitope Database (IEDB) (http://tools.iedb.org/) [30]. Two servers, HLApred (http://www.imtech.res.in/raghava/hlapred) and MHC II binding prediction tool (http://tools.immuneepitope.org/analyze/html/mhc_II_bindin g.html) in IEDB analysis resource were used for the detection of CD4+ T cell epitopes [31,32]. All the classical propensity scale methods in IEDB i.e. Kolaskar and Tongaonkar antigenicity scale, Emini surface accessibility prediction, Parker hydrophilicity prediction, Karplus and Schulz flexibility prediction, Bepipred linear epitope prediction and Chou and Fashman beta turn prediction (http://tools.immuneepitope.org/bcell/) were utilized for the identification of linear B cell epitopes. Conformational B cell epitopes were predicted on the basis of solvent-accessibility and flexibility by ElliPro from IEDB analysis resource (http://tools.immuneepitope.org/ellipro/) which generates 2D score plot, 3D image and residual score for each epitope [33].

### IFN-γ induction capacity prediction and docking analysis:

To assess the IFN-γ induction capacity, both the HPAG1_0576 protein and predicted B-cell linear epitope (19-35) were fed to an online tool namely IFNepitope (http://crdd.osdd.net/raghava/ifnepitope/scan.php) [13]. This program generates all the possible overlapping peptides at window length of 20 and predicts IFN epitope in these overlapping peptides as well as rank them on the basis of SVM score. To perform molecular docking between epitope and receptor, 3D structure of two IFN-γ receptor alpha chains (PDB id: 1fyh and 1jrh) were retrieved from RCSB Protein Data Bank (www.rcsb.org). The protein-protein docking of IFN-γ receptor and HPAG1_0576 was performed by PatchDock server and the 10 best outputs of the PatchDock [34] were fed to FireDock for refinement [35]. The most suitable IFN receptor was selected based on the docking pose with the lowest global energy.

## Results

### Structure prediction:Characterization of primary and secondary structure:

Primary structure of the target protein was revealed by ProtParam and the computed parameters proposed that, the amino acid Leucine was most prevalent in the protein sequence that suggests a preference of alpha helices in its 3D structure (Table 1). The prediction outcomes for protein secondary structure generated by SOPMA found alpha helices (59.38%) to be most frequent which also supports the ProtParam interpretation (Table 5) [18].

### Homology modeling:

**Figure 1:**
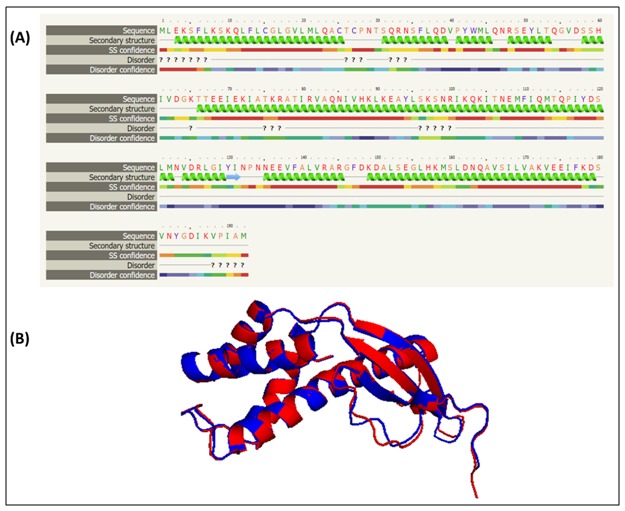
Structural alignment between the template and HPAG1_0576. (A) Secondary structure alignment between 2wcr and HPAG1_0576 constructed via Phyre2. (B) The PyMOL view of the superimposed structures in RaptorX in which red color represents HPAG1_0576 structure, while the blue for template (2wcr).

After analyzing the results of homology modeling it was found that, Phyre2 generated 20 possible models for the target protein based on alignment with different templates. The best model was obtained with the highest scoring template (PDB id: 2wcr) which stands for Tip-α protein that induces expression of TNF-α in B cell and promotes tumor activities and thus results in gastric cancer [7]. The model was predicted with 100% confidence, 14% disorder and 76% alignment coverage. Figure 1 displays the secondary and 3D structure alignment of the modeled protein with its template.

### Refinement,quality assessment, energy minimization and visualization of the model:

**Figure 2:**
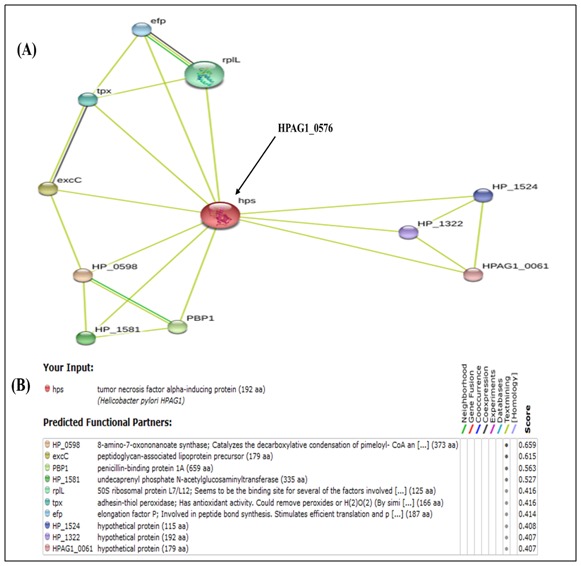
PPI network of HPAG1_0576 detected via STRING. Different line colors represent the types of evidence for the association. (A) The evidence view of the interacting network. (B) The predicted functional partners of the protein HPAG1_0576.

**Figure 3:**
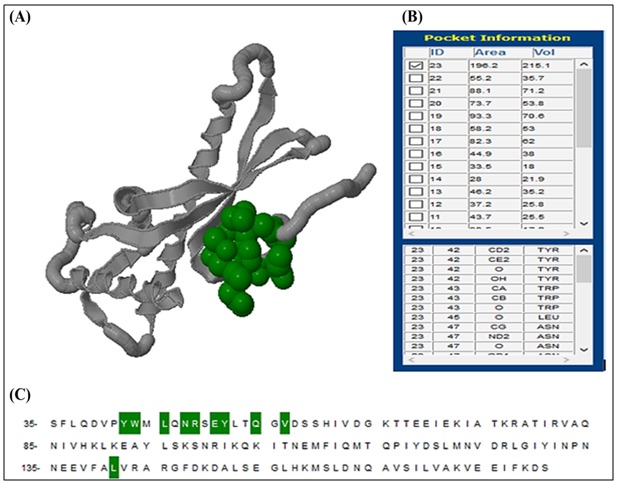
Active site analysis by CastP. (A) The 3D structure of the best active site. (B) The area and the volume for different pockets of HPAG1_0576. (C) Conserved amino acid position from 35 to 141 with residues occurring in active sites (green color).

ModRefiner refined the selected model by detecting high resolution protein structure with an RMSD 0.237 and TMscore 0.9972. The backbone conformation, internal consistency and reliability of the protein were evaluated by PROCHECK which created Ramachandran plot (Table 3) with acceptable amino acid distribution for this model (Figure 1). Verify 3D and ERRAT analysis showed the overall quality values of 0.64 and 96.35 respectively (Figure 2). The Z score values by ProSA and QMEAN has been depicted in Figure 3.

### Functional annotation:

The metadata server ProFunc made a general assessment using gene ontology terms defining the protein as DNA binding and involved in cellular processes. InterProScan found one motif match against Pfam database and it was TNF-α inducing protein of Helicobacter. Blast against PDB and UniProt found 25 and 50 matching sequences respectively. In addition, ProFunc output identified 664 matching folds, two nests, one enzyme active site and twenty reverse templates from the structure of HPAG1_0576.

### PPI network analysis:

At medium confidence (0.400), PPI network analysis by STRING showed that, HPAG1_0576 was highly similar to hps (TNF-α inducing protein from H. pylori HPAG1) with highest bitscore and e-value of 400 and 1e-141 respectively. Figure 2 represents the PPI network of hps and demonstrates that, the target protein interacts with 10 other proteins. The highest confidence was 0.659 and observed with 8-amino-7-oxononanoate synthase (HP_0598) which catalyzes the decarboxylative condensation of pimeloyl- CoA and L-alanine to produce 8-amino-7-oxononanoate (AON), coenzyme A and/or converts 2-amino-3-ketobutyrate to glycine and acetyl-CoA. Other interacting partners were: a peptidoglycan-associated lipoprotein precursor, a penicillinbinding protein 1A, undecaprenyl phosphate N-acetyl glucosaminyl transferase, a 50S ribosomal protein L7/L12 which seems to be the binding site for several of the factors involved in protein synthesis and appears to be essential for accurate translation, an elongation factor P which is involved in peptide bond synthesis and other three hypothetical proteins.

### Active site analysis:

CastP predicted 23 active sites of the modeled HPAG1_0576 which are associated with binding pockets within the protein. The best model which is usually considered standard was chosen on the basis of area, volume and conserved residues in the pockets. The largest pocket (pocket 23) had an area and volume of 196.2 and 215.1 Å respectively. The residues occurring in this pocket were TYR42, TRP43, LEU45, ASN47, ARG48, GLU50, TYR51, GLN54, VAL56 and LEU141 (Figure 3).

### T-cell epitope prediction:

VaxiJen predicted that, HPAG1_0576 was a probable antigen. Therefore, NetCTL predicted 57 different CD8+ T cell epitopes of the protein according to all MHC (A1-B62) supertypes among which 4 most potential epitopes with high combinatorial scores were selected. The interacting MHC-I alleles with each of the four epitopes at affinity IC50 < 200 are shown in Table 1. It also shows epitope conservancy and the combined scores of epitope-HLA interactions. MHC class II binding prediction tool and HLApred retrieved five common epitopes that are strong binders to HLADRB1* 01:01, HLA-DRB1*04:01, HLA-DRB1*07:01and HLADRB1* 11:01. Similar human epitopes were eliminated and having an IC50 value less than 50 were selected [36]. The epitopes FLCGLGVLM, FLQDVPYWM, FLKSKQLFL, FALVRARGF and IKVAQNIVH were identified as potential CD4+ T-cell epitopes and which could elicit an immune response.

### B-cell epitope prediction:

**Figure 4:**
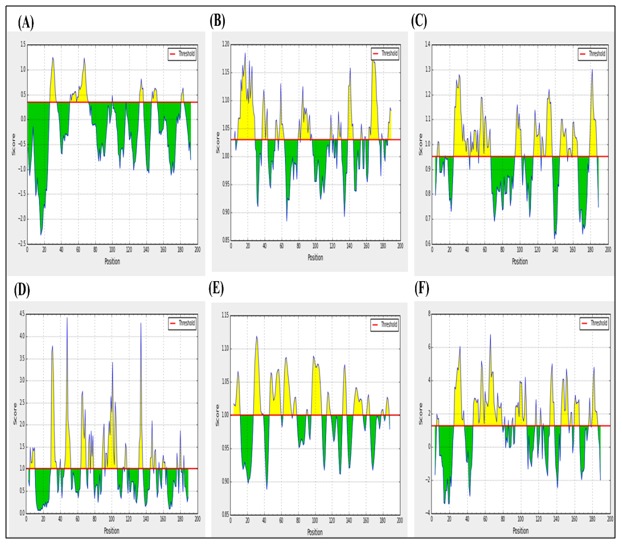
B cell linear epitope prediction of HPAG1_0576 protein using, (A) Bepipred linear epitope prediction. (B) Kolaskar and Tongaonkar antigenicity prediction. (C) Chou and Fasman beta turn prediction. (D) Emini surface accessibility prediction. (E) Karplus and Schulz flexibility prediction. (F) Parker hydrophilicity prediction. Here, yellow regions in the plot represent potential B cell epitopes having scores above their threshold values.

**Figure 5:**
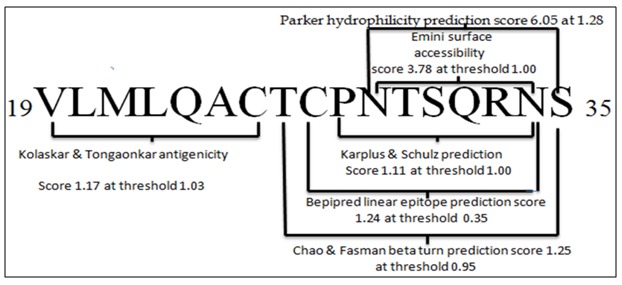
Outcome of combined B cell linear epitope prediction from HPAG1_0576. The scores for all different IEDB scales are shown at correspondent threshold value.

**Figure 6:**
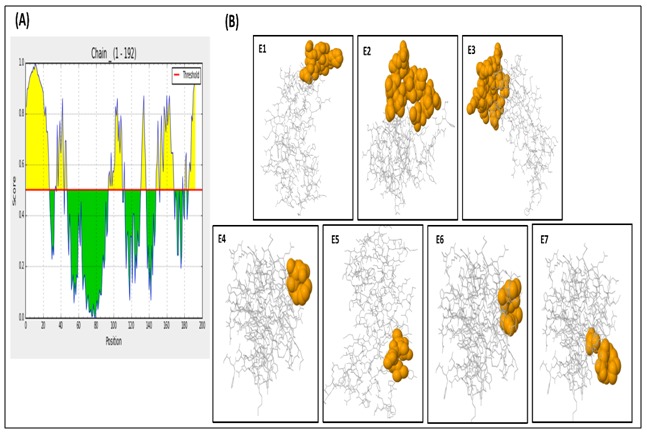
B cell discontinuous epitopes of HPAG1_0576 predicted by ElliPro. (A) X and Y axis represents the residue number and scores respectively. Yellow regions in the plot represent potential B cell epitopes having a score above the threshold 0.5. (B) Jmol visualization of the predicted epitopes, where antibody chains are represented in white and epitopes in orange.

Epitopes those satisfied the threshold values for all five IEDB scales with highest antigenic propensities were considered to evoke potent B cell response and found to reside within19 to 35 residues spanning the sequence (Figure 4). Figure 5 depicts the combined linear epitope with spanning peptides, highest antigenicity scores and their corresponding threshold values. Ellipro predicted seven conformational epitopes as well as their residual specifications and scores that are summarized in Table 2. Among them, SFLKSKQL is the most potential with the highest score 0.971. Figure 6 represents the 2D score chart and 3D images of the predicted epitopes shown as ball-and-stick models.

### Prediction of IFN-γ induction and docking analysis:

**Figure 7:**
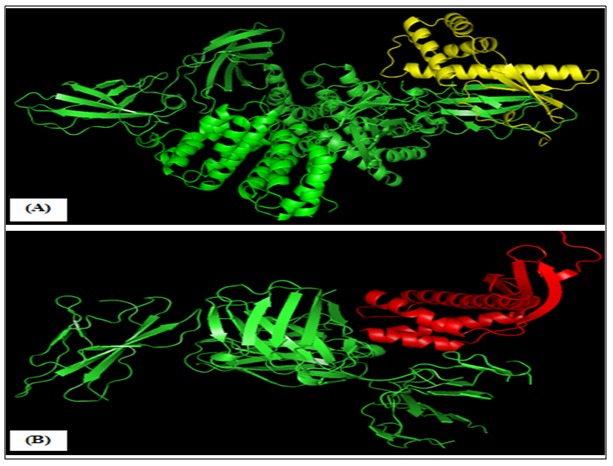
Best docking pose of HPAG1_0576 protein with IFN-γ receptor alpha chain (1FNGR1). Post docking refinement by FireDock the best interaction was visualized by PyMOL. (A) Interaction between ligand (HPAG1_0576 in yellow color) and receptor (1fyh in green color). (B) Interaction between ligand (HPAG1_0576 in red color) and receptor (1jrh in green color).

The findings of IFNepitope program suggests that, both the target protein and predicted B cell linear epitope had great probability to release of IFN-γ with a positive score. Within the region between 64 to 83 (GKTTEEIEKIATKRATIRVA) of HPAG1_0576 showed the maximum SVM score of 1.52, while the predicted B cell linear epitope had hybrid (motif+SVM) score of 3.0. The rigid and symmetric docking of HPAG1_0576 protein with the IFN-γ receptor was done in PatchDock and first 10 docking candidates were submitted to FireDock, which refines and scores them according to an energy function. The best docking pose showed an energetically favorable interaction between HPAG1_0576 and IFN-γ receptor alpha chain (Figure 7). The docking and post docking refinement results ranked on global energy of the best solution has been shown in Table 3, where the global energy (GE) is the binding energy of a solution. Transformation refers to 3D transformation with 3 rotational angles and 3 translational parameters and applied on the ligand molecule. Here score means geometric shape complementary score; area is approximate interface area of the complex; Vdw is Van der Walls; ACE means the contribution of the atomic contact energy (ACE) to the global binding energy and HB is the contribution of hydrogen bonds to global binding energy.

## Discussion

The present study identified a HP, HPAG1_0576 from H. pylori strain HPAG1, which showed a strong homology with a member of Tip-α superfamily. Since the crystal structure of this HP is unavailable, the study is proposing a structural model constructed via homology modeling using the crystal structure of a TNF-α inducer protein (PDB id: 2wcr) as a template. Initially the physicochemical characterization was done by ExPASy's ProtParam tool and the prediction results are the deciding factors for the hydrophilicity, stability and function of the protein [37]. Findings from SOPMA revealed that, the protein has a high helices percentage in its structure, which can facilitate protein folding by providing more flexibility to the structure, thus protein interactions might be increased [5]. Moreover, an abundance of coiled regions contributes to higher stability and conservation of the protein structure [37]. Phyre2 built the 3D structure of HPAG1_0576 with 100% confidence, which indicates that, the core of the protein is modeled at high accuracy. For extremely high accurate model, the percent identity between sequence and template should be above 30-40%; hence for the constructed model in this study, the identity was found 98%. The quality of the structural alignment was confirmed by RaptorX (Figure 1B), that produced template modeling (TM) score 0.973 and RMSD 0.91 which denotes that the structures are almost identical because identical structures score 1 whereas highly similar models have a TM-score >0.7 [19].

**Figure 8:**
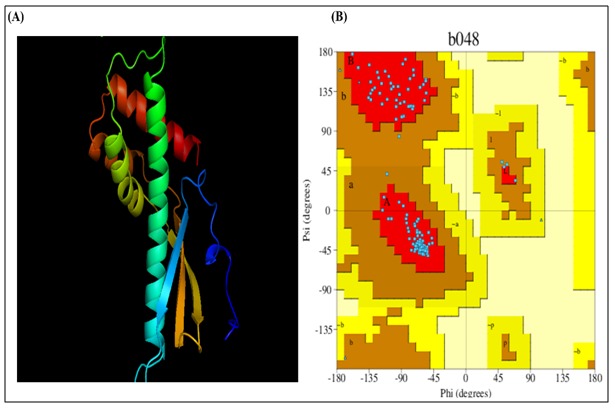
Structural analysis of HPAG1_0576. (A) The cartoon view of the refined 3D structure in PyMOL. (B) Ramachandran plot showing 97.1 % of amino acid residues in the core region (red color).

**Figure 9:**
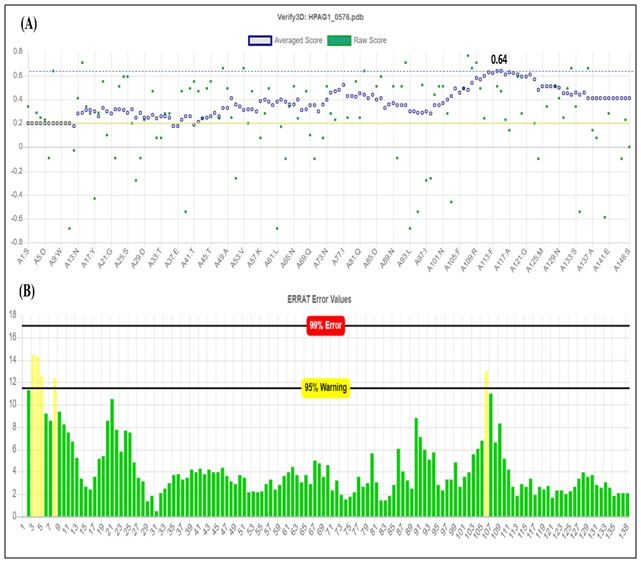
Overall quality evaluation by Verify3D and ERRAT. (A) Verify3D prediction outcome showing 97.26% residues having 3D- 1D average score > 0.2 and highest score obtained 0.64. (B) ERRAToutput with quality factor 96.35. Two lines on the error axis indicate the confidence within which the regions that exceed the error value can be rejected.

**Figure 10:**
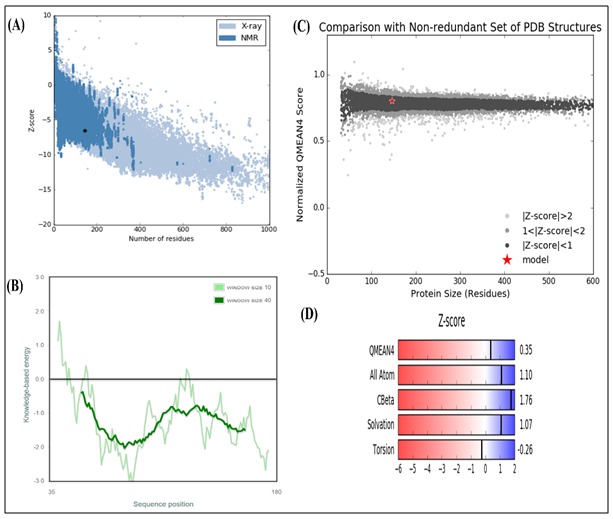
Calculation of Z score. (A) The ProSA Z-score of modeled HPAG1_0576 was obtained -6.5 (highlighted as black large dot). (B) The ProSA energy plot showing local model quality. (C) Estimated absolute quality plot from QMEAN analysis, the red star in the dark region indicates the protein of interest. Models are extpected to lie in this region to be considered good. (D) QMEAN4 value as well as individual Zscores.

The resolution required for protein applications such as ligand screening and understanding reaction mechanism was obtained by refining the model using ModRefiner. The distribution of the residues in Ramachandran plot supports good stereo chemical quality of the model (Figure 8B) [38]. The 3D-1D average score 0.64 obtained fromVerify3D indicates a better environmental profile of the model Figure 9A [37]. The overall quality factor 96.35, obtained by ERRAT denotes the percentage of residues for which the calculated error value cannot exceed the 95% rejection limit Figure 9B [23]. The Z score obtained from ProSA for the obtained model was −6.5 Figure 10A, which was well fitted to the range that is typical for proteins of similar size. The local model quality is shown in the energy plot Figure 10B and minimum values in the plot account for nativity and stability of the molecules [5,39]. The QMEAN4 score for the protein was obtained 0.35 Figure 10D, which was in the range of estimated global model reliability score between 0 to 1 [38]. Hence, the protein of interest is in the dark region of the absolute model quality plot with a global score 0.7 which also supported the quality of the model [39]. Individual Z values for parameters such as C-β interaction energy, all atom interaction, solvation and torsion can also be observed in the plot Figure 10C.

The significant similarity of the modeled HPAG1_0576 with its template indicates its likely function as Tip-α. Though, no single method is reliable in terms of correct prediction [37], therefore the meta server ProFunc was used and the structure was found to contain 664 matching folds among which four had certain matches with PDB codes 3gio, 2wcr, 3guq and 3vnc. One enzyme active site template that was identified in possible matches is E. coli heat-labile entero toxin with bound galactose (PDB id: 1lta) with 37.5% sequence identity. The function of 'reverse' template method is to break the target into many templates which are then scanned against a set of representative structures in PDB. Among the 370 auto-generated templates, certain matches were observed again with 2wcr, 3gio, and 3vnc confirming the Phyre2 prediction of the protein as Tip-α [27,40].

Detailed study of protein-protein interactions network will help to elucidate the signaling pathways of human diseases and their drug targets as well [41]. From STRING analysis (Figure 3), the nearest interaction of HPAG1_0576 was observed with another HP of H. pylori, HP_0598 which is 8-amino-7-oxononanoate synthase. Other interacting partners are: a peptido-glycan associated lipoprotein precursor (excC), a penicillin-binding protein 1A (PBP1), an undecaprenyl phosphate N-acetyl glucosaminyl transferase (HP_1581), a 50S ribosomal protein L7/L12 (rplL), an adhesinthiol peroxidase (tpx) having antioxidant activity, an elongation factor P (efp) involved in peptide bond synthesis and other three hypothetical proteins of Helicobacter. Shape and size parameters of protein pockets and cavities are important for structure-based ligand designing. The top pocket in the CastP output list is the largest and considered as standard (Figure 4A).

Since the protein is found to stimulate the immune system by activating NF-κB pathway, it is considered as highly immunogenic and proved so by VaxiJen server. To design an effective peptide antigen, the recommended length of peptide sequences should be within 8-22 amino acids. In this study, the continuous B cell epitope VLMLQACTCPNTSQRNS (position 19- 35) was 17 residues long and the discontinuous epitope SFLKSKQL (5-12) was 8 residues long. The study also focused on searching natural epitopes that would stimulate both CD8+ and CD4+ T cell response, to mediate a more balanced response in the prevention of disease prognosis. Four potential CD8+ T cell epitopes (Table 1) have been identified so far among which, FALVRARGF is the most potential with highest I pMHC immunogenicity score, this epitope was also predicted as CD4+ T cell epitope with high immunogenicity. The high level of epitope conservancy is much more important because Tip-α has a higher tendency towards mutation, hence epitope conservancy was found 100% for both [14,42].

## Conclusion

It is of interest to study the structure to function information for antigenic characterization of a hypothetical protein designated as HPAG1_0576 from Helicobacter pylori HPAG1. We report that, the structural model of HPAG1_0576 shows it as a cytoplasmic protein with a Tip-α domain having unique DNA binding function. We also discuss the linear and conformational antigenic regions in the protein for potential consideration as a vaccine candidate. Further experimental studies are required to validate the predicted epitopes. Future studies are in progress to experimentally validate the data found from this study and to use the structural and functional information of the given model to identify novel ligands for new drug discovery.

## Figures and Tables

**Table 1:** Predicted CD8+ T-cell epitopes with total scores and interacting MHC-1 alleles

CD8+ epitope	NetCTL combined score	Interacting MHC-1 allele with an affinity of IC50>200	Epitope conservancy	I pMHC immunogenicity prediction score
FLKSKQLFL	1.16; A2	HLA-A*02:01(0.11)	100%	-0.49647
1.69; B8	HLA-B*08:01 (0.11)
HLA-C*01:02 (-1.78)
HLA-C*07:04 (-1.98)
FIQMTQPIY	0.93; A1	HLA-A*29:02 (0.91)	100%	-0.21714
0.92; A26	HLA-B*15:02 (0.52)
1.05; B62	HLA-B*35:01 (0.65)
HLA-C*07:04 (-1.93)
HLA-C*14:02 (-0.59)
FALVRARGF	1.64; B8	HLA-B*52:01(-1.56)	100%	0.18865
0.86; B62	HLA-B*55:01(-1.60)
HLA-C*01:02 (-1.53)
HLA-C*02:02 (-0.91)
HLA-C*02:09 (-0.91)
HLA-C*03:02 (1.24)
HLA-C*03:04 (0.69)
HLA-C*07:04 (-1.6)
MLQNRSEYL	1.53; B8	HLA-A*02:01 (0.15)	100%	-0.0671
0.78; B62	HLA-C*07:01 (-1.64)
HLA-C*07:04 (-2.14)
HLA-C*14:02 (-0.68)

**Table 2:** Predicted discontinuous epitopes of HPAG1_0576 protein by Ellipro tools

Epitope No.	Residues	Number of residues	Score
1	_:S5, _:F6, _:L7, _:K8, _:S9, _:K10, _:Q11, _:L12	8	0.971
2	_:M1, _:L2, _:E3, _:F13, _:L14, _:C15, _:G16, _:L17, _:G18, _:V19, _:L20, _:M21, _:L22	13	0.898
3	_:Y94, _:L95, _:S96, _:K97, _:S98, _:N99, _:R100, _:I101, _:K102, _:Q103, _:K104, _:I105, _:T106, _:N107, _:E108, _:M109, _:Q112, _:D148, _:K149, _:D150, _:A151, _:S153, _:E154, _:G155, _:L156, _:H157, _:K158, _:M159, _:S160, _:L161, _:D162, _:N163, _:Q164, _:A165, _:V166, _:S167, _:I168	37	0.686
4	_:R33, _:N34, _:S35, _:F36, _:L37, _:Q38, _:D39, _:V40, _:P41, _:Y42, _:W43, _:I131, _:N132, _:P133, _:N134, _:N135, _:E136, _:E137, _:G184, _:D185, _:I186, _:K187, _:V188, _:P189, _:I190, _:A191, _:M192	27	0.673
5	_:Q23, _:A24, _:C25, _:T26, _:C27, _:P28	6	0.642
6	_:L45, _:Q46, _:N47, _:S49	4	0.536
7	_:S180, _:V181, _:N182, _:Y183	4	0.507

**Table 3:** and post docking analysis results for ligand HPAG1_0576 and IFN-γ receptor alpha chain interaction

Receptor	Score	Area	Transformation	Global energy	Attractive VdW	Repulsive VdW	ACE	HB
1fyh	16462	2114.1	-0.20 -0.12 0.29 -29.94 28.34 -73.69	-6.02	-30.6	18.03	14.66	-3.88
1jrh	15834	2369.9	3.09 -0.50 -2.77 54.76 13.80 95.14	-3.09	-5.79	2.57	-1.99	0

**Table 4:** Physicochemical properties of the target protein (HPAG1_0576) calculated by ProtParam

Parameters	Calculated values
Molecular weight	21851.32
Theoretical Pi	8.36
Total number of negatively charged residues (Asp + Glu)	22
Total number of positively charged residues (Arg + Lys)	24
Extinction coefficients, assuming all pairs of Cys residues form cystines	14565
Extinction coefficients, assuming all Cys residues are reduced	14440
Instability index	49.85
Aliphatic index	95.94
Grand average of hydropathicity (GRAVY)	-0.22

**Table 5:** Secondary structural elements of HPAG1_0576 calculated by SOPMA

Secondary structure	Residue percentage
Alpha helix(Hh)	59.38% (114)
Extended strand (Ee)	14.06% (27)
Beta turn (Tt)	8.85% (17)
Random coil (Cc)	17.71% (34)
310 helix	0.00%
Pi helix	0.00%
Beta bridge	0.00%
Bend region	0.00%

**Table 6:** Observed G factor scores and percent distribution of the amino acid residues in HPAG1_0576 created by Ramachandran plot

Ramachandran plot statistics	% of distribution	G factor score
% of Residues in most favored regions	97.10%	Dihedral angles 0.7
% of Residues in additional allowed regions	2.90%	Main chain covalent forces 0.34
% of Residues in generously allowed regions	0.00%	Overall average 0.17
% of Residues in disallowed regions	0.00%
